# The potential effects of the United States abortion ruling on global embryo/foetal and stem cell research

**DOI:** 10.1093/lifemedi/lnac052

**Published:** 2022-11-18

**Authors:** Zhenyu Xiao, Jianwei Lv, Siqi Zhao, Rosario Isasi, Xinwei Xie, Lei Dong, Yaojin Peng

**Affiliations:** School of Life Science, Beijing Institute of Technology, Beijing 100081, China; Beijing Institute for Stem Cell and Regenerative Medicine, Beijing 100101, China; Beijing Institute for Stem Cell and Regenerative Medicine, Beijing 100101, China; State Key Laboratory of Stem Cell and Reproductive Biology, Institute of Zoology, Chinese Academy of Sciences, Beijing 100101, China; State Key Laboratory of Stem Cell and Reproductive Biology, Institute of Zoology, Chinese Academy of Sciences, Beijing 100101, China; University of the Chinese Academy of Sciences, Beijing 100049, China; Dr John T. Macdonald Foundation Department of Human Genetics and John P. Hussman Institute for Human Genomics, University of Miami Miller School of Medicine, Miami, FL 3310, USA; School of Life Science, Beijing Institute of Technology, Beijing 100081, China; School of Life Science, Beijing Institute of Technology, Beijing 100081, China; Beijing Institute for Stem Cell and Regenerative Medicine, Beijing 100101, China; State Key Laboratory of Stem Cell and Reproductive Biology, Institute of Zoology, Chinese Academy of Sciences, Beijing 100101, China; University of the Chinese Academy of Sciences, Beijing 100049, China

On 24 June 2022, the United States (US) Supreme Court ruled in *Dobbs v. Jackson Women’s Health Organization* (*Dobbs* case), overturning a half century of legal precedent in *Roev. Wade* (1973), revoking the constitutional right to abortion and leaving the decision on the legality of abortion to the discretion of individual states. Although this ruling focussed on the constitutional right to abortion, it is highly likely to affect the future direction of embryonic/foetal and stem cell research policies in the USA and even other jurisdictions worldwide, leaving the biomedical field fraughted with uncertainty in the long run.

Debates on abortion and the ethics of embryo/foetal research have converged to become a key force influencing US embryo/foetal research policy. Although the USA has had no legal ban at the federal level, since 1996, each government regulates embryo research mainly by formulating its federal funding policy according to the Dickey–Wicker Amendment and consistent with its stance to abortion. At the state level, embryo research policies vary widely between states. Some fund human embryo research [particularly human embryonic stem cell (hESC) research], whereas others ban all human embryo research [1]. In terms of federal policy of foetal research, it has varied depending on each government’s stance on abortion as well. For instance, based on its opposition to abortion, the Trump Administration eliminated some federally funded research that relied on foetal tissue from elective abortions and began to regulate the rest more strictly; on the contrary, based on its support for abortion, the Biden Administration reversed the ban. Most importantly, influenced by the *Dobbs* case, states have recently moved to adopt strict legislation regulating access to abortion services (ranging from severe restrictions to outright bans).

In addition, the abortion debate to some extent has profoundly shaped the development of embryo research policies in various jurisdictions generally. In particular, jurisdictions influenced by religious teaching tend to be more anti-abortion, are more sensitive to the moral status of embryos, and tend to prohibit or severely restrict embryo research, such as Italy, Russia, Turkey, and Austria. In Germany, the Federal Constitutional Court ruled in 1975 and 1993 that the law on abortion was unconstitutional, granting the unborn foetus the right to life. Germany explicitly prohibits embryo research in any sense. Jurisdictions, such as UK, have actively sought-after biomedical advancement by taking a relatively liberal approach, allowing embryo research and human embryonic stem cell (hESC) research with certain restrictive conditions, such as the 14-day rule (i.e., human embryos should not be cultured *in vitro* beyond 14 days post-fertilization or formation of the primitive streak, whichever occurs first). Furthermore, some jurisdictions, including China, Japan, and the Republic of Korea, do not consider embryos to have full moral status, and are therefore tolerant of abortion and do not impose too many restrictions on embryo research.

Overall, the issues of abortion and embryo research are intertwined, with abortion policy one of the major influencing factors in the formulation of embryo research policies. Both topics ask the fundamental question of when life begins or whether an embryo is human. The *Dobbs* case demonstrates that abortion is a profound moral issue and there have always been conflicting views. Those who believe human life begins at conception consider abortion killing an innocent human life. Others argue that abortion should be allowed in certain circumstances, but the limits have been disputed. Therefore, it is vital and valuable to evaluate the long-term and far-reaching effects of the *Dobbs* case on embryonic and related research (Fig. 1).

**Figure 1.**
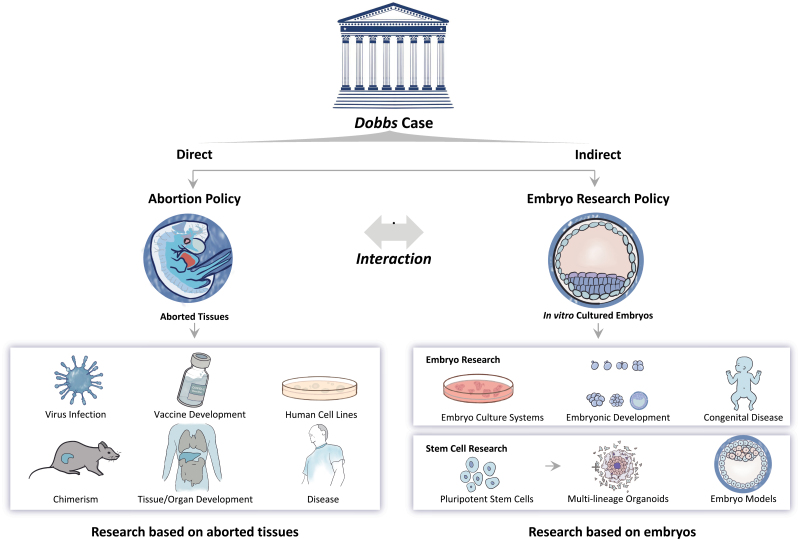
The long-term and far-reaching effects of the *Dobbs* case on embryonic and related research.

The *Dobbs* case will first affect abortion policy and then gradually affect research using aborted embryo/foetal tissues. The *Dobbs* case has increased the number of anti-abortion states for the foreseeable future, which will undoubtedly reduce the supply of aborted foetus tissue, and thereby undermining the development of related research. Currently, many cells or substances derived from aborted tissue have been used in the biomedical research field. For instance, cells isolated from aborted tissues are used to make nutritive feeders, and astrocytes from the aborted foetus brain are used to nourish induced pluripotent stem cell neurons [2]. Dopaminergic neurons derived from aborted foetuses are transplanted into patients’ brains to treat Parkinson’s disease [3]. Some research requires the use of foetal tissue to make humanized animal models [4]. Since the humanized system is not genetic, animal models must be created repeatedly, leading to the constant demand for foetal tissue.

The indirect effects of the *Dobbs* case on attitudes toward the policy on embryo research may undermine the development of research based on *in vitro* cultured human embryos. Over the past few decades, we have learned about the development of complex organs and systems and have gained insight into the aetiology and cellular basis of multiple congenital disorders by culturing human embryos *in vitro* within a narrow time window (due to the 14-day rule) [5]. However, a growing number of anti-abortion groups may use the *Dobbs* case ruling as an opportunity to push for further federal and state government restrictions on human embryo-­related research, making public and private sector funding for human embryo-related research more restrictive. Moreover, it will affect the careers of embryo research scientists who live in jurisdictions with stricter regulations, meaning they will be forced to conduct scientific tourism should they wish to conduct embryo-related research. This could lead to an embryo research brain drain in the *pro-life* regions of the USA. Finally, the *Dobbs* case will increase the confidence of opponents of embryo research, which may also prompt a challenge to the rationality of the 14-day rule indirectly, bringing a great deal of uncertainty to the future of medical research.

The short-term effects of the *Dobbs* case on stem cell research is likely to be limited because a number of stem cell lines derived from embryo/foetal tissues have been established. The long-term effects, however, may be more damaging. Reduced sources of aborted foetal tissue and potential restrictions on embryo research will inevitably affect the production of new human cell lines or stem cell lines that are still playing significant roles in several biomedical fields, such as vaccinology, infectious diseases, and regenerative medicine. For instance, stem cell-based embryo models and multi-lineage organoids, based on hESCs or other extraembryonic stem cells such as human trophoblast stem cells, are providing new insight at different angles for understanding human development, fertility, and the later stages of human life. Hundreds of stem cell lines have already been established but the new are still required to construct more bona fide embryo or organ models.

The *Dobbs* case may potentially have a ripple effect on other jurisdictions. Jurisdictions that share similar cultural values to the USA may impose more restrictions on embryo research. The uncertainty of the future embryo research policy in the USA will likely have a more significant effect on centrist jurisdictions. For conservative jurisdictions such as Germany and Austria, their embryo research policies will not be affected much. For countries such as UK, which prioritize advancements in biomedicine, their embryo research policies are expected to remain liberal. In addition, East Asian jurisdictions such as China, the Republic of Korea, and Japan are expected to be less affected because their cultural difference insulates them from changes in the USA.

Future changes to embryonic, foetal, and related stem cell research policies will greatly affect relevant research in various scientific fields. To some extent, the *Dobbs* case will likely generate more policy uncertainty for these fields of research and may even change trends in technological development. Consequently, it is vital and necessary for the scientific community to recognize the potential effects of the *Dobbs* case on biomedical research. Certain precautions may be taken to mitigate possible adverse effects. For instance, organizing and participating in international symposiums on embryo research policy formation and issuing relevant international declarations would be meaningful. Additionally, the scientific community may also actively speak to the public and policymakers, strive for their support, and lobby for balanced laws and regulations in the field. Only in this way can we responsibly promote the healthy development and future welfare of humankind.

## References

[1] Matthews KR, Morali D. Can we do that here? An analysis of US federal and state policies guiding human embryo and embryoid research. J Law Biosci 2022;9:lsac014. 35692936 10.1093/jlb/lsac014 PMC9183789

[2] Freed CR, Zhou W, Breeze RE. Dopamine cell transplantation for Parkinson’s disease: the importance of controlled clinical trials. Neurotherapeutics 2011;8:549–61. 21997523 10.1007/s13311-011-0082-9 PMC3250289

[3] Madrazo I, Kopyov O, Ávila-Rodríguez MA, et al. Transplantation of human neural progenitor cells (NPC) into putamina of parkinsonian patients: a case series study, safety and efficacy four years after surgery. Cell Transplant 2019;28:269–85. 30574805 10.1177/0963689718820271 PMC6425108

[4] Shultz LD, Brehm MA, Garcia-Martinez JV, et al. Humanized mice for immune system investigation: progress, promise and challenges. Nat Rev Immunol 2012;12:786–98. 23059428 10.1038/nri3311 PMC3749872

[5] Xiang L, Yin Y, Zheng Y, et al. A developmental landscape of 3D-cultured human pre-gastrulation embryos. Nature 2020;577:537–42. 31830756 10.1038/s41586-019-1875-y

